# Supplementary material for: Sorting at embryonic boundaries requires high heterotypic interfacial tension
Source: Nat Commun. 2017 Jul 31;8:157. doi: 10.1038/s41467-017-00146-x (PMC5537356; doi:10.1038/s41467-017-00146-x)
Supplement: Supplementary file 2 — Supplementary Software 1 [file 41467_2017_146_MOESM2_ESM.zip › PottsModel/SrcPottsModel/doc/model/Lattice.html]

Lattice


JavaScript is disabled on your browser.


Skip navigation links


- Overview
- Package
- Class
- Use
- Tree
- Deprecated
- Index
- Help

- Prev Class
- Next Class

- Frames
- No Frames

- All Classes

- Summary:
- Nested |
- Field |
- Constr |
- Method

- Detail:
- Field |
- Constr |
- Method


model

## Class Lattice

- java.lang.Object
- - mvc.AObservable
  - - model.Lattice

- All Implemented Interfaces:
  :   java.lang.Iterable<Pixel>, KnowsConstants, IObservable, Observer

  ---

    

  ```
  public class Lattice
  extends AObservable
  implements Observer, java.lang.Iterable<Pixel>, KnowsConstants
  ```

- - ### Nested Class Summary

    Nested Classes

    | Modifier and Type | Class and Description |
    | --- | --- |
    | `class` | `Lattice.PixelIterator` |
    | `static class` | `Lattice.State` |
    | `static class` | `Lattice.Type` |
  - ### Field Summary

    Fields

    | Modifier and Type | Field and Description |
    | --- | --- |
    | `int` | `aHeight` |
    | `int` | `aWidth` |
    | `static java.lang.String` | `cDefaultConfigFile` |
    | `static java.lang.String` | `cNotificationSource` |
  - ### Constructor Summary

    Constructors

    Constructor and Description

    `Lattice(Constants pConstants)`

    `Lattice(Constants pConstants,
    int[][][] rawLattice)`

    Initialize grid with specified pixel IDs.
  - ### Method Summary

    All Methods Static Methods Instance Methods Concrete Methods

    | Modifier and Type | Method and Description |
    | --- | --- |
    | `PottsCanvas` | `getCanvas()` |
    | `java.util.Iterator<Cell>` | `getCellIterator()` |
    | `CellSet` | `getCells()` |
    | `Constants` | `getConstants()` |
    | `java.lang.String` | `getNotificationSource()` |
    | `Pixel` | `getPixel(int pX, int pY)` |
    | `java.awt.Color[][]` | `getPixelColors()` |
    | `Lattice.PixelIterator` | `getPixelIterator()` |
    | `IPositionManager` | `getPositionManager()` |
    | `static Lattice` | `inverse(Constants pConstants)` |
    | `java.util.Iterator<Pixel>` | `iterator()` |
    | `java.lang.String` | `toString()` |
    | `void` | `update(javax.management.Notification pNotification)` Determines what an observer should do upon notification that the observed object has changed. |
    | `void` | `updateAllPixelParameters()` |
    | `boolean` | `withinBounds(int pX, int pY)` |

    - ### Methods inherited from class mvc.AObservable

      `addObserver, getNotificationSequenceNumber, notifyObservers, removeAllObservers, removeObserver`
    - ### Methods inherited from class java.lang.Object

      `equals, getClass, hashCode, notify, notifyAll, wait, wait, wait`
    - ### Methods inherited from interface java.lang.Iterable

      `forEach, spliterator`

- - ### Field Detail


    - #### cNotificationSource

      ```
      public static final java.lang.String cNotificationSource
      ```

      See Also:
      :   Constant Field Values


    - #### cDefaultConfigFile

      ```
      public static final java.lang.String cDefaultConfigFile
      ```

      See Also:
      :   Constant Field Values


    - #### aWidth

      ```
      public final int aWidth
      ```


    - #### aHeight

      ```
      public final int aHeight
      ```
  - ### Constructor Detail


    - #### Lattice

      ```
      public Lattice(Constants pConstants)
      ```


    - #### Lattice

      ```
      public Lattice(Constants pConstants,
                     int[][][] rawLattice)
      ```

      Initialize grid with specified pixel IDs.

      Parameters:
      :   `pPixelIDs` -
  - ### Method Detail


    - #### inverse

      ```
      public static Lattice inverse(Constants pConstants)
      ```


    - #### getPixelColors

      ```
      public java.awt.Color[][] getPixelColors()
      ```


    - #### toString

      ```
      public java.lang.String toString()
      ```

      Overrides:
      :   `toString` in class `java.lang.Object`


    - #### withinBounds

      ```
      public boolean withinBounds(int pX,
                                  int pY)
      ```


    - #### getPixel

      ```
      public Pixel getPixel(int pX,
                            int pY)
      ```

      Parameters:
      :   `pX` - x coordinate
      :   `pY` - y coordinate

      Returns:
      :   APixel located at the given coordinates if within bounds, else
          null


    - #### getCanvas

      ```
      public PottsCanvas getCanvas()
      ```


    - #### update

      ```
      public void update(javax.management.Notification pNotification)
      ```

      Description copied from interface: `Observer`

      Determines what an observer should do upon notification that the observed object has changed.

      Specified by:
      :   `update` in interface `Observer`

      Parameters:
      :   `pNotification` - : Notification passed by the object being observed.


    - #### getNotificationSource

      ```
      public java.lang.String getNotificationSource()
      ```

      Specified by:
      :   `getNotificationSource` in interface `IObservable`

      Overrides:
      :   `getNotificationSource` in class `AObservable`


    - #### getPixelIterator

      ```
      public Lattice.PixelIterator getPixelIterator()
      ```


    - #### getCellIterator

      ```
      public java.util.Iterator<Cell> getCellIterator()
      ```


    - #### getPositionManager

      ```
      public IPositionManager getPositionManager()
      ```


    - #### updateAllPixelParameters

      ```
      public void updateAllPixelParameters()
      ```


    - #### iterator

      ```
      public java.util.Iterator<Pixel> iterator()
      ```

      Specified by:
      :   `iterator` in interface `java.lang.Iterable<Pixel>`


    - #### getConstants

      ```
      public Constants getConstants()
      ```

      Specified by:
      :   `getConstants` in interface `KnowsConstants`


    - #### getCells

      ```
      public CellSet getCells()
      ```


Skip navigation links


- Overview
- Package
- Class
- Use
- Tree
- Deprecated
- Index
- Help

- Prev Class
- Next Class

- Frames
- No Frames

- All Classes

- Summary:
- Nested |
- Field |
- Constr |
- Method

- Detail:
- Field |
- Constr |
- Method
